# A Reverse Genetics System for the Israeli Acute Paralysis Virus and Chronic Bee Paralysis Virus

**DOI:** 10.3390/ijms21051742

**Published:** 2020-03-04

**Authors:** Sa Yang, Hongxia Zhao, Yanchun Deng, Shuai Deng, Xinling Wang, Qingyun Diao, Chunsheng Hou

**Affiliations:** 1 Institute of Apicultural Research, Chinese Academy of Agricultural Sciences, Beijing 100093, China; sayang1994@163.com (S.Y.); 18852861125@163.com (Y.D.); Shdeng11@163.com (S.D.); wangxinlingjiayou@126.com (X.W.); dqyun1@126.com (Q.D.); 2 Key Laboratory of Pollinating Insect Biology, Ministry of Agriculture and Rural Affairs, Beijing 100093, China; 3 Guangdong Key Laboratory of Animal Conservation and Resource Utilization, Guangdong Public Laboratory of Wild Animal Conservation and Utilization, Guangdong Institute of Applied Biological Resources, Guangzhou 510260, China; hxzh110@126.com; 4 Graduate School of Chinese Academy of Agricultural Sciences, Beijing 100093, China

**Keywords:** reverse genetics, IAPV, CBPV, infectious clone, *Apis mellifera*

## Abstract

Honey bee viruses are associated with honey bee colony decline. Israeli acute paralysis virus (IAPV) is considered to have a strong impact on honey bee survival. Phylogenetic analysis of the viral genomes from several regions of the world showed that various IAPV lineages had substantial differences in virulence. Chronic bee paralysis virus (CBPV), another important honey bee virus, can induce two significantly different symptoms. However, the infection characteristics and pathogenesis of IAPV and CBPV have not been completely elucidated. Here, we constructed infectious clones of IAPV and CBPV using a universal vector to provide a basis for studying their replication and pathogenesis. Infectious IAPV and CBPV were rescued from molecular clones of IAPV and CBPV genomes, respectively, that induced typical paralysis symptoms. The replication levels and expression proteins of IAPV and CBPV in progeny virus production were confirmed by qPCR and Western blot. Our results will allow further dissection of the role of each gene in the context of viral infection while helping to study viral pathogenesis and develop antiviral drugs using reverse genetics systems.

## 1. Introduction

Honey bees not only provide bee products to mankind but also supply an essential pollination service to terrestrial ecosystems [1]. In China in particular a variety of crops and wild plants must be pollinated [2]. In recent years, the decline of honey bee populations has led to concern as colony losses have been associated with viruses, honey bee pathogens about which little is known [3]. Small RNA viruses of the honey bee were considered as the main cause of honey bee colony decline investigated in America and a few European countries [4]. Increasing reports of new viruses infecting honey bees are still emerging [5], although over 20 viruses have been confirmed to have effects on honey bee health. 

Of these viruses, Israeli acute paralysis virus (IAPV) has been considered as a major contributor to colony collapse disorder (CCD) [6], although further studies have failed to confirm a direct causal link with CCD. IAPV was first identified and characterized in 2007, and it was found that a typical sign of IAPV was characterized by a paralyzed body and an inability to fly, progressing to death in front of the beehive and eventually resulting in the collapse of the entire colonies in a short time [7]. Then, it was found that IAPV not only occurred widely in many regions worldwide [8] but also had a high prevalence in both *Apis mellifera* and *Apis cerana* in China [9] as well as other hymenopteran insects such as *Vespula vulgaris* [10]. In addition, it can be transmitted by *Varroa destructor*, a parasite that poses a severe threat to the health and survival of honey bees around the world. The interaction between the Varroa mite and the virus not only reduced host immunity but also elevated the level of IAPV and led to a rapid decline in colony populations [11,12]. IAPV was frequently involved in covert infections or co-infections at lower titers within a colony [13,14], but obvious symptoms were observed when IAPV was rescued and injected into healthy pupae [14]. Indeed, a report showed that IAPV can be rescued at a low level under natural conditions and has greater infectivity and virulence in healthy honey bees [15]. Moreover, IAPV is present in almost every colony throughout the whole year, but the titer is higher in winter [15]. Thus, IAPV can slow honey bee population growth and result in large losses in the following spring. 

An important reason why it is difficult to detect and prevent IAPV is that the biological and molecular characterizations of IAPV, Kashmir bee virus (KBV), and acute bee paralysis virus (ABPV) are quite similar [14], but there are distinct differences in their genomes [16]. Additionally, recombination often occurs in different segments of a certain IAPV genome, in the RNA or RNA sequence between IAPV and other dicistroviruses/IAPV and nonviral RNAs, and in the exchange between nonretroviruses and their hosts [17]. Moreover, virion protein 1 (VP1), one of the structural proteins of IAPV, does not contain a hydrophobic pocket that functions as a site for binding antiviral compounds in other picornaviruses [18]. This evidence demonstrated that we might clearly characterize and prevent infections if an infectious clone were to become available. 

Chronic bee paralysis virus (CBPV), another infectious and contagious virus, is mainly composed of two segments, RNA 1 and RNA 2, which can cause two significant symptoms [19]. One symptom is referred to as “bloated abdomen”, caused by the onset of dysentery which cannot be excreted. The other is known as “black robbers” or “hairless black syndrome”. Generally, CBPV can infect individuals of all developmental stages at a lower level [20]. However, the titer of CBPV is elevated when it encounters extrinsic stressors. For example, *Nosema ceranae* is one important parasite of the honey bee that can weaken the strong colony and result in poor spring establishment, reducing population growth [21]. Thiamethoxam is considered as a crucial environmental agent used widely to control pests in agriculture crops [22]. When co-infection on individual bees with CBPV and *N. ceranae* was performed, the replication level of CBPV increased [23]. Similar results were observed when bees were exposed to thiamethoxam [22]. However, the function of each segment and the interaction between CBPV and other stressors remain unclear.

Although intense efforts have been made to prevent IAPV and CBPV, an accurate characterization of the interactive relationship between viruses and the host cannot be accomplished due to the lack of in vitro honey bee viral cell culture systems. In fact, several studies have attempted to address this subject, but have not been successful in practice [24,25,26,27,28]. So far, all reports on honey bee viral cells with different bee viruses using different mediums based on different tissues including *A. cerana* have achieved the expected results [29,30,31,32]. Therefore, constructing infectious clones has become a feasible way to study the pathogenesis of honey bee viruses. An infectious transcript of black queen cell virus (BQCV) was obtained by amplification of the entire genome sequence [33]. Lamp et al. [34] constructed a deformed wing virus (DWV) infectious clone that was 10,164 bp in length based on the pBR322 vector and identified its infectiousness and pathogenesis. Recently, a molecular clone of CBPV was made based on T vector and confirmed that 5′-cap structures were required for viral replication [35].

However, an infectious clone has not been developed for IAPV. Thus, in the present study, we constructed a full-length infectious clone of IAPV using the pACYC177 vector, successfully identified its pathogenesis in adult bees in vitro, and proposed an effective model for the study of honey bee RNA viruses in the absence of a cell culture system. In addition, we built an infectious clone for CBPV using the same vector. Thus, we have used efficient methods to construct an infectious clone of honey bee viruses from natural and synthetic RNA using a universal vector.

## 2. Results

### 2.1. Construction and Characterization of Infectious Clones

**Figure 1:**
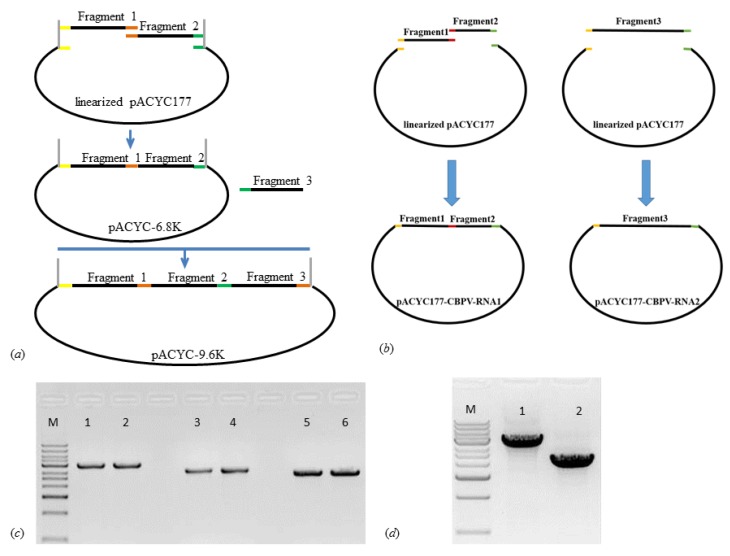
Schematic diagrams of the infectious clones for Israeli acute paralysis virus (IAPV) and chronic bee paralysis virus (CBPV). The steps for construction the infectious clones of IAPV (**a**) and CBPV (**b**). PCR amplification target fragments of IAPV (**c**) and CBPV (**d**). M indicates the DNA ladder. Numbers 1–6 in Figure c indicate fragment 1 (lanes 1 and 2), fragment 2 (lanes 3 and 4), and fragment 3 (lanes 5 and 6) of IAPV. Numbers 1–2 in Figure d represent fragment 1 and 2 of RNA 1 and fragment 3 of RNA 2 of CBPV.

The strategy for building a full-length infectious clone of IAPV and CBPV is schematically shown in Figure 1a,b. To generate the IAPV cDNA clone, the full-length IAPV was amplified from bee samples collected in Beijing apiaries by using RT-PCR. The three individual fragments of DNA were assembled into the low-copy plasmid vector pACYC177 (Figure 1c). After several bacterial passages, the plasmid sequence was confirmed as being identical to the expected sequence. The constructed IAPV infectious clone was 9599 nt in length (Accession number: MG599488) and was longer than any other published full-length IAPV genome sequence. Similarly, two precise full-length fragments of RNA 1 and RNA 2 of CBPV were integrated into pACYC177 (Figure 1d). RNA 1 and RNA 2 were found to be 3674 nt and 2305 nt in length, respectively (Accession numbers: MF175173 and MF175174). In addition, we purified and sequenced IAPV and CBPV from bees injected with their infectious clones to confirm that the sequences of progeny virus were consistent with those of infectious clones of IAPV and CBPV. 

### 2.2. Genome Sequences of Infectious IAPV and CBPV

**Figure 2:**
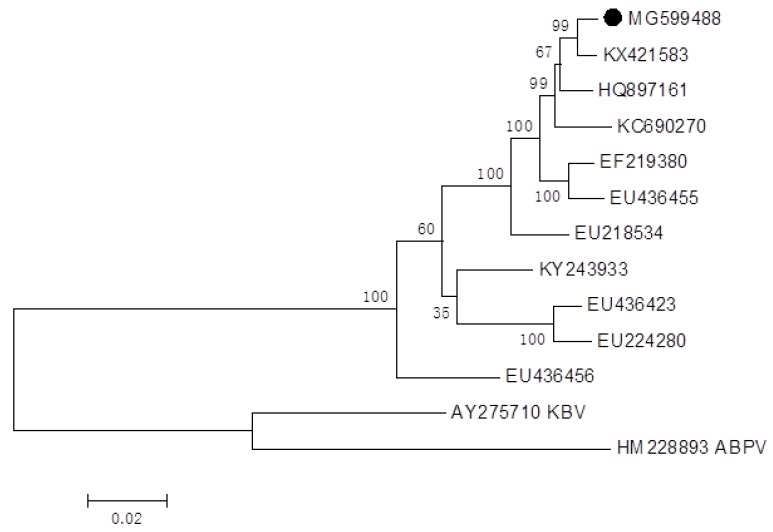
Phylogenetic analysis of the infectious clone sequence of IAPV. The black dot indicates the obtained IAPV in the current study (Number MG599488). Bootstrap values are indicated on each node as a result of the 1000 replicates calculated.

A phylogenetic analysis showed that our infectious clone of IAPV was similar to the published IAPV sequences from the NCBI database (Figure 2). IAPV was close to Chinese strains KX421583 and quite different from the Australia strain (EU436456). We did not perform a phylogenetic analysis on the RdRp of CBPV because Seitz et al. 2019 [35] created a phylogenetic tree using the RdRp genes involving our infectious clone CBPV (MF175173 for RNA 1). As shown in their results, current CBPV formed a separate subgroup, which was similar to strains from France, Belgium, and Spain.

### 2.3. Assess the Infectivity of Infectious Clones

**Figure 3:**
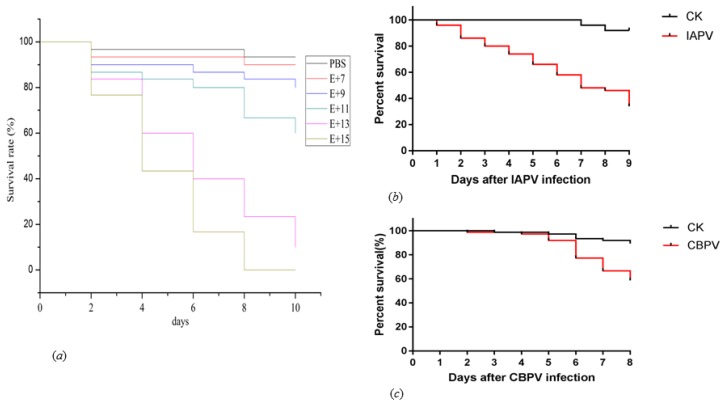
Effects of infectious clones of IAPV and CBPV on honey bees survival rates. The survival rates of honey bees in the control groups and those injected with five different concentrations of the infectious clone of IAPV: 1 × 10^7^, 10^9^, 10^11^, 10^13^, and 10^15^ genomic copies (**a**). The survival rates of honey bees in the control groups and those injected with 10^12^ genomic copies of the infectious clones of IAPV (**b**) and CBPV (**c**). CK: blank control.

The linearized IAPV and CBPV templates were used for in vitro RNA transcription using T7 RNA polymerase, and the resulting full-length IAPV and CBPV transcripts were injected into the abdomen of healthy adult bees to observe their viral pathogenesis. First, we used five serially diluted concentrations of IAPV to determine the infectivity based on the previous studies [36,37]. Doses of 1 × 10^7^, 10^9^, 10^11^, 10^13^, and 10^15^ genomic copies were injected into healthy adult bees, and the mortality was recorded each day. It was found that honey bee mortality was induced by the infectious clone of IAPV. Based on this observation, we decided to use a dose of 1 × 10^12^ to perform the following experiment according to the survival rate (Figure 3a). Then, we assessed the in vivo replication ability of IAPV and CBPV. After 3 or 4 days post-infection, most of the adult bees infected with an RNA transcript showed typical paralysis symptoms of IAPV and CBPV, which suggested that the RNA transcripts were infectious. None of the bees infected with phosphate buffered saline (PBS) or a blank control showed paralysis, and the survival rate exceeded 90% even after 8 days. As shown in Figure 3b,c, honey bee mortality of infectious IAPV and CBPV gradually increased from day 1 to day 8 compared with the control groups. When comparing IAPV with CBPV, we found the mortality of IAPV was higher than that of CBPV.

### 2.4. Detection the Pathogenesis of Infectious Clones

**Figure 4:**
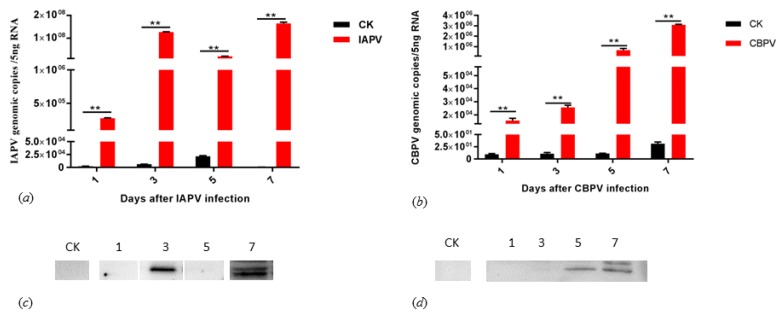
Quantification and identification of the level in bees and pathogenesis on bees injected with infectious clones of IAPV and CBPV. Expression levels of bees infected with infectious IAPV (**a**) and CBPV (**b**) at days 1, 3, 5, and 7. Detection of the IAPV virion protein 2, VP2 (**c**) and CBPV structural protein (SP) (**d**) of IAPV- and CBPV- infected bees at days 1, 3, 5, and 7. Note: Numbers 1, 3, 5, and 7 indicate infectious IAPV and CBPV bee samples collected on days 1, 3, 5, and 7. CK: blank control. Asterisks indicate significant differences (** *p* < 0.01).

To assess the replications of the infectious clones of IAPV and CBPV, we tested the IAPV and CBPV genomic copies on bees injected with the infectious clone and PBS at days 1, 3, 5, and 7, respectively. As shown in Figure 4a, the general trend for IAPV titers steadily increased from day 1 to day 7 with the exception of day 3, peaking at 1.65 × 10^8^ genome copies, returning to 6.30 × 10^6^ at day 8, and decreasing afterwards (data not shown). However, the titers of CBPV steadily increased until day 7, peaking at 3.08 × 10^6^ genomic copies (Figure 4b). To further confirm the expression of RNA transcripts, we divided the bee samples into two parts; one was used to quantify the genome copy number, and the other was used for detecting the presence of IAPV capsid protein VP2 by Western blot using an anti-VP2 antiserum as previously described [15] as well as the CBPV SP protein. An immunoblotting analysis indicated that the target proteins of IAPV and CBPV were present on days 3 and 7 of IAPV-infected bees and days 5 and 7 of CBPV-infected bees, but we did not detect any target proteins in any control bees (Figure 4c,d). Two days after infection, IAPV-infected bees started to show apparent symptoms such as paralysis and trembling. Similarly, CBPV-infected bees showed the typical signs of infection 4 days after being infected.

## 3. Discussion

Reverse genetics is a powerful tool for studying the pathogenesis of viruses, especially for single-strand positive RNA viruses. The first IAPV infectious clone was constructed in the present study, with typical symptoms shown and pathogenesis confirmed. We also constructed an infectious clone for CBPV using the same vector with that of IAPV. More importantly, we provided a universal method for constructing infectious clone of honey bee viruses.

Biological cloning methods in existing cell culture systems have not been established for honey bee viruses. Although reports deem cell systems for bee viruses feasible [30,38], a large gap between theory and application still exists. An infection model was necessary to test candidate genetic factors associated with viral pathogenesis. Reverse genetics are a key technique for illuminating viral infection characteristics and the roles of viral genes. Here, we introduced the first plasmid based on a reverse genetics system for the IAPV genome. This infectious clone is longer than the available sequences and has an identity with most reported sequences, especially in the conserved part. We used a clone vector that differed from those used for DWV [34] and BQCV [33]. Although we also attempted to construct the full genome sequence using the T vector and a similar method, we failed. This failure might have resulted from the differences in the genus among IAPV, DWV, and BQCV. In addition, we used the pACYC177 vector to construct infectious IAPV and CBPV, and suggest this vector might provide a universal tool for infectious honey bee viruses and for the easy construction of infectious clones of a single virus. This current study was thus not only successful in constructing an infectious IAPV but also supplied a practical model in vitro for constructing infectious clones of honey bee viruses.

Furthermore, bees infected by these two infectious clones displayed visible paralysis as previously described. They lost the ability to fly and then became paralyzed, with death after several days [7]. Compared with infectious DWV, the pathogenicity of the IAPV infectious clone had a lower mortality rate of 78% than that of the DWV-A clone at day 4 [34]. As shown in Figure 4, although the titers of infectious clones of IAPV and CBPV were increased from days 1 to 7, the viral proteins were present at days 3 and 7, and days 5 and 7, respectively. These results were consistent with the previous studies in which the infected bee showed typical paralysis symptoms [37,39]. The reason why only the RNA transcript can play a viral function of pathogenesis is because of the structural characteristics of their genome. IAPV has a single-stranded positive RNA genome of about ~9.3 kb in length, which contains two nonoverlapping open reading frames (ORFs) [7]. These two ORFs are translated by an independent host factor [40,41]. Although CBPV has two major segments, RNA 1 and RNA 2, they employ a similar method to translate structural proteins [42]. Recent studies showed that the injection of the two CBPV RNA segments was sufficient to initiate viral replication in honey bees [19]. In addition, they all have a capped structure in the 5’ end of their genomes. 5’-cap structures are essential for the efficient gene expression of mRNA molecules and for protecting RNA molecules from degradation by cellular exonucleases [40].

IAPV has a longstanding presence in managed honey bees [43]. Although IAPV is not consistently tied to CCD, its ability to cause increased mortality in honey bees has been firmly identified. Here, we reported the first plausible infectious clone of IAPV and confirmed that IAPV has a similar pathogenesis to reported strains from different geographical regions [20]. More importantly, it provides a possible way to characterize the infection dynamics of IAPV if several viruses are co-infected within one bee. This allows us to focus on a severe threat even if it does not predominate at the start of infection [32]. Hence, we can study a single virus in detail, or even co-infection with several viruses, and virulence factors or virulence associated with gene alterations during an infection in the honey bee. The present study provides a better starting point for addressing the molecular mechanisms of virus pathogenesis and will also serve as a platform for heterologous gene expression to explore virus–host interactions. 

## 4. Materials and Methods 

### 4.1. Honey Bee Samples 

IAPV-positive adult worker honey bees (*A. mellifera*) showing visible paralysis symptoms were collected from Beijing apiaries in 2015 and 2016. Significantly crawling CBPV-positive adult worker bees were sampled from Hubei province apiaries in 2017. To confirm the pathogenesis of the IAPV and CBPV infectious clones, we collected seemingly healthy bees from the experimental apiary of the Institute of Apicultural Research (IAR). Honey bee samples were collected from inside each colony. Newly emerged bees were obtained from brood frames taken from the experimental honey bee hives and kept in an incubator at 30 °C and a 60% relative humidity (RH) for approximately 12 h until use for injecting RNA transcripts. We confirmed the absence of the eight common viruses before the experiment was performed as previously described [44].

### 4.2. Screening for the Presence of Common Viruses

A single brood frame with emerging worker bees (*A. mellifera*) was removed from experimental colonies of the IAR and kept in an incubator at 34 °C with 80% relative humidity overnight. Newly emerged bees were obtained after 24 h and used to detect the presence of eight common viruses—Israeli acute paralysis virus (IAPV), Sacbrood virus (SBV), deformed wing virus (DWV), black queen cell virus (BQCV), chronic bee paralysis virus (CBPV), acute bee paralysis virus (ABPV), *Varroa destructor* virus (DWV-B), and Chinese sacbrood virus (CSBV) (Table S1). The bee samples uninfected with the common viruses mentioned above were placed in groups of 30 bees per small cage for molecular clone RNA treatment. 

### 4.3. Virus Purification

To purify the virus, IAPV was screened for predominance in the samples, and the absence of other common viruses was also verified. Approximately 50 adult honey bees were collected from five colonies of three apiaries in Beijing in which paralysis symptoms were found. Positive samples were used to extract crude virus, which was used to infect healthy honey bee pupae. After three days, IAPV was purified as described by de Mirada et al. [14]. Briefly, an IAPV crude suspension was injected into the abdomen of white-eyed worker honey bee pupae (*A. mellifera*). The injected pupae were raised in an incubator at 34 °C for 3 days. Approximately 300 bee pupae were crushed in 40 mL of 0.01 M PBS buffer with 2 mL diethyldithiocarbamic acid. The mixture was centrifuged at 2500 g for 30 min. The supernatant was centrifuged at 15,000 g for 4 h. The precipitated virus was resuspended in 1 mL Brjo buffer with a sucrose gradient and centrifuged at 12,500 g for 4 h. The virus fraction was recovered in a CsCl gradient and was centrifuged at 17,000 g for 24 h. Likewise, CBPV purification employed similar protocol with IAPV as described by Olivier et al. [20].

### 4.4. RNA Extraction and cDNA Synthesis

IAPV and CBPV viral genomic RNA were isolated from a purified suspension with a QIAamp viral RNA Mini Kit (Qiagen, Germany). The samples were centrifuged for 5 min at 9313 g and the supernatant was used for RNA extraction according to the manufacturer’s instructions. The total RNA was dissolved in 20 µL of elution buffer and stored at −80 °C until use. 

The purified RNA was used to synthesize the first-strand cDNA with an oligo (dT) primer using GoScript Reverse Transcriptase (Promega, Madison, WI, USA). Three pairs of primers were designed to amplify three overlapping nucleotide sequence fragments of the full sequence according to the IAPV genome sequences deposited in GenBank (No. EF219380) using Phusion High-Fidelity PCR Master Mix (Thermo Scientific, USA). PCR amplification was performed as follows: 30 s at 98 °C, 10 s at 98 °C, 30 s at 61 °C (55 °C for CBPV), 90 s at 72 °C for 35 cycles, and 10 min at 72 °C for an extra extension. The PCR products were subsequently electrophoresed in a 1.5% agarose gel containing GoldView (SBS Genetech Corp. Ltd., Beijing, China); then purified products were cloned into the pEASY-Blunt cloning vector (TransGen Biotech, Beijing, China) and transformed into competent *Escherichia coli* cells (HB101) for sequencing [35]. For CBPV, two pairs and one pair primers for RNA 1 and RNA 2, respectively, were used to amplify the full sequence of both fragments. The sequences, orientation and location of the primers, as well as the expected product sizes, are shown in Table S2. 

### 4.5. Construction of the Infectious Clone and RNA Synthesis

To facilitate the cloning of the entire genome length cDNA of IAPV, a low copy vector, pACYC177 (CWBio, Beijing, China), was used to generate a stable clone. Three fragments of IAPV were amplified to generate 2.8 kbp, 3.8 kbp, and 3.1 kbp fragments with high-fidelity Phusion HiFi PCR Master Mix (NEB, Ipswich, MA, USA) using specific primers (Table S2). The first fragment introduced the sequence of the T7 promoter (TAATACGACTCACTATAGGG) at the 5′ end to facilitate in vitro transcription. The full-length infectious clone was completed using homologous recombination with a ClonExpressMultiS One-Step Cloning Kit (Vazyme Biotech Co., Nanjing, China). The pACYC177 vector with *E. coli* as the host bacterium (Promega, Madison, WI, USA) was used for all cloning experiments using standard protocols. All necessary enzymes, including the Goscript reverse transcription system and TransStart FastPfu Fly DNA Polymerase, were purchased from TransGen Biotech (Beijing, China), and the reactions were performed according to the manufacturer’s instructions. Purification of all DNA fragments was performed using a QIA Quick gel extraction kit (Qiagen, Hamburg, Germany).The resultant constructs were transformed into *E. coli* and selected on chloramphenicol-containing media. A plasmid containing the expected IAPV fragments was sent to Shanghai Sangon Bio-Tech (Shanghai, China) for verification by sequencing. No differences in the nucleotide sequences were observed between the progeny IAPV and the infectious cloned IAPV. 

For in vitro RNA synthesis, each stable plasmid pACYC177 containing the full-length cDNA was extracted with a Plasmid MiniPrep Kit (TransGen Biotech, Beijing, China) and linearized with Dpn I. We first amplified the full-length IAPV on the positive plasmid and confirmed it with T7-13FL/IAPV9613R as forward and reverse primers. Then, we treated and recycled it with Dpn I and LiCl, respectively. Subsequently, the transcription reaction was performed with a HiScribe T7 Quick High Yield RNA Synthesis Kit (NEB, Ipswich, MA, USA) according to the manufacturer’s recommended protocol and the RNA was purified with an EasyPure RNA Kit (TransGen Biotech, Beijing, China). The concentration was measured using a NanoDrop spectrophotometer (Thermo Scientific, Beijing, China), and the RNA quality was confirmed in a 1.2% gel using formaldehyde denaturing electrophoresis and elution in 30 µL RNase-free water. Viral RNA from transcript-derived IAPV was extracted from injected honey bees using a QIAamp Viral RNA mini Kit (Qiagen, Hamburg, Germany) according to the manufacturer’s instructions. Purified RNA was treated with reverse transcriptase using standard PCR amplification conditions to amplify the target fragments. The PCR products were purified and sequenced, then used for subsequent experiments to confirm sequence identity with the infectious clone. For construction of the infectious CBPV clone, the general protocol and chemical agents were similar to those of IAPV. 

### 4.6. Assessment the Viral Growth 

We determined the experimental concentrations of IAPV for subsequent experiments. Naturally infected honey bees have a range of IAPV abundances. Given that the covert IAPV infection concentration was approximately 10^5^–10^7^ [15], we tested 10^7^, 10^9^, 10^11^, 10^13^, and 10^15^ genomic copies in each 2 µL of a dilution series. All experiments were run in three replicates and each replicate contained three individuals.

Newly emerging bees were selected to identify those negative for IAPV and were screened for the common viruses mentioned above. Treated bees were injected with 2 µL of purified synthetic RNA (approximately 1 × 10^12^ genome copies) with the third to fourth integument of the honey bee abdomen and infected with a Hamilton syringe (702) (Hamilton, Switzerland). The control group was injected with PBS. No further virus was added. After injection, these groups were transferred to an incubator at 30 °C and 60% relative humidity, and mortality was observed and recorded daily.

### 4.7. Quantification of the Replication Level of Rescue Virus

Individual bees from each group were cut into two parts through the middle of the back; half of the bee was used to quantify the transcriptional levels by qPCR and the other half for detecting the signal of VP2 and SP of IAPV and CBPV by Western blot, respectively. cDNA produced by conventional RT-PCR was subjected to real-time quantitative PCR using a LineGene 9600 instrument (Bioer, Hangzhou, China). The primers used for IAPV and the housekeeping gene (*actin*) were as follows: IAPV forward 5’-CCAGCCGTGAAACATGTTCTTACC-3’ and reverse 5’-ACATAGTTGCACGCCAATACGAGAAC-3’ were used to amplify a 226-bp fragment [15]; and housekeeping gene forward primers β-actin 5’-TTGTATGCCAACACTGTCCTTT-3’ and reverse β-actin 5’-TGGCGCGATGATCTTAATTT-3’ were used to amplify a reference gene fragment [45]. For quantification of CBPV, we used 5’-CAACCTGCCTCAACACAG-3’ and 5’-AATCTGGCAAGGTTGACTGG-3’ as forward and reverse primers to amplify a 296-bp fragment. Quantitative PCR was performed using the KAPA SYBR FASTqPCR kit Master Mix (Sigma-Aldrich, USA) according to the manufacturer’s instructions. The cycling protocol was 95 °C for 3 min followed by 40 cycles of 95 °C for 3 s, 60 °C for 30 s, and 72 °C for 20 s. Standard curves were prepared by performing real-time qPCR with serial 10-fold dilutions of known concentrations of the IAPV and CBPV-specific amplicons. The melting curve dissociation was analyzed to confirm each amplicon. The concentration of nonspecific primer amplification was measured by performing the RT reaction in the absence of added primers followed by specific-primer quantitative real-time PCR and was found to be negligible under present working conditions. The results were analyzed using 9600 Plus Software. 

### 4.8. Protein Extraction and Western Blot

Protein was extracted from bee halves as mentioned above. The bees were ground with steel beads in PBS containing 0.2% diethyl dithiocarbamate (DDC) and 1 µM phenylmethanesulfonyl fluoride (PMSF). An additional 200 µL extraction buffer was added to the resulting protein extract and quickly centrifuged. Then, the supernatant was centrifuged at 15 °C at 9313 g for 10 min, and 200 µL chloroform were added, with centrifuging again at 4 °C at 9313 g for 10 min. The protein was resuspended in double-distilled water (DDW) and diluted with 2× concentrated sample buffer (100 mM Tris–HCl, pH 6.8, 4% sodium dodecyl sulfate (SDS), 17% glycerol, and 0.8 M 2-mercaptoethanol), boiled for 3 min, and subjected to sodium dodecyl sulfate-polyacrylamide gel electrophoresis (SDS-PAGE) at a constant voltage (110 V). The polypeptides were separated on 12% SDS-PAGE prior to electrophoretic transfer to nitrocellulose membranes (Pall Corporation, New York, USA). For colloidal Coomassie staining, the gels were fixed for 30 min in 0.85% o-phosphoric acid/20% methanol and then stained overnight in 20% methanol according to the manufacturer’s instructions. The gels were destained in 25% methanol. To detect IAPV and CBPV antigens, the blots were blocked with 5% skim milk powder and probed with different rabbit polyclonal antibodies against IAPV VP2 and CBPV SP synthesized by Hangzhou HuaanBiotech (Hangzhou, China). The blots were developed using the Amersham ECL plus reagent (GE Healthcare, Chalfont St Giles, UK) and exposed to a C-Digit scanner (Licor, Lincoln, USA) and imaged by photography.

### 4.9. Statistical Analysis

The average longevity among the different treatment groups was statistically tested with a Kaplan–Meier survival analysis with log rank test statistics using the GraphPad Prism 5 software. The variation in the IAPV and CBPV titers between the treatments was analyzed using one-way ANOVA. The qPCR data were tested for normality with a Kolmogorov-Smirnov test and log-transformed if they did not show normality. Statistical analyses were done using the GraphPad Prism 5.

## Supplementary Materials

Supplementary materials can be found at https://www.mdpi.com/1422-0067/21/5/1742/s1.
