# Supplementary material for: A Reverse Genetics System for the Israeli Acute Paralysis Virus and Chronic Bee Paralysis Virus
Source: Int J Mol Sci. 2020 Mar 4;21(5):1742. doi: 10.3390/ijms21051742 (PMC7084666; doi:10.3390/ijms21051742)
Supplement: Supplementary file 1 [file ijms-21-01742-s001.zip › ijms-710611-supplementary final/Table S1.docx]

**Table S1.** Primers used for PCR detection common honey bee viruses

| Virus | Primer（5′-3′） | Annealing temperature (℃) Target fragment (bp) |
| --- | --- | --- |
| IAPV | AGACACCAATCACGGACCTCAC AGATTTGTCTGTCTCCCAGTGCAC | 55 474 |
| SBV | ATATACGGTGCGAGAACTGC CTCGGTAATAACGCCACTGT | 56 879 |
| ABPV | TTATGTGTCAGAGACTGTAT GCTCCTATTGCTCGGTTTTTC | 55 900 |
| BQCV | TGGTCAGCTCCCACTACCTTAAAC GCAACAAGAAGAAACGTAAACCAC | 57 700 |
| CBPV | TCAGACACCGAATCTGATTATTG ACTACTAGAAACTCGTCGCTTCG | 55 570 |
| DWV-B | CATAGCGAATTACGGTGCAA GAGGGGTCCCTACTCTACCG | 55 200 |
| DWV | CTTACTCTGCCGTCGCCCA CCGTTAGGAACTCATTATCGCG | 55 376 |
| CSBV | CCTGGGAAGTTTGCTAGTATTTACG CCTATCACATCCATCTGGGTCAG | 55 161 |
