# Supplementary material for: A Reverse Genetics System for the Israeli Acute Paralysis Virus and Chronic Bee Paralysis Virus
Source: Int J Mol Sci. 2020 Mar 4;21(5):1742. doi: 10.3390/ijms21051742 (PMC7084666; doi:10.3390/ijms21051742)
Supplement: Supplementary file 1 [file ijms-21-01742-s001.zip › ijms-710611-supplementary final/Table S2.docx]

**Table S2. Primers for amplification target fragments of IAPV and CBPV**

| Virus | Abbreviation | Primer sequence (5′–3′) | Description |
| --- | --- | --- | --- |
| IAPV | T7-13FL | TAATACGACTCACTATAGGGACTTTTATGTCCCTACGTACAATTTTCGCCGAAATT | Amplification of fragment 1 |
|  | IAPV3790R | CTTAGGAGTATTTACAAGAGATTCTGGGTC |  |
|  | IAPV3771F | CTCTTGTAAATACTCCTAAGGTTGAAGCTTTTGC | Amplification of fragment 2 |
|  | IAPV6840R | GCCGATGAAGTATCCTGAGCC |  |
|  | IAPV6822F | CTCAGGATACTTCATCGGCTAGGAAC | Amplification of fragment 3 |
|  | IAPV9613recR | TCAGTATTGACTGCACCGGTTTTTTTTTTTTTTTTTAAATTTACCTAATTCGAAAATTTTG |  |
|  | pACYC1905F | CCCTATAGTGAGTCGTATTAAGTAATACAAGGGGTGTTATGA | Amplification of linearized vector pACYC177 |
|  | IA6820-P1580R | GCTCAGGATACTTCATCGGCATACTGGCTTACTATGTTGG |  |
|  | IAPV6840R | ---------------------------------------- | Amplification of linearized vector pACYC-6.8k |
|  | pACYC1580R | ACCGGTGCAGTCAATACTGAATACTGGCTTACTATGTTGG |  |
|  | IAPV3400F | CTGGAAATGGCTATGCTCTGATGG | Identification of recombinant vector pACYC-6.8k and pACYC-IAPV |
|  | IAPV6840R | ---------------------------------------- |  |
|  | pACYC1567F | GACACCCTCATCAGTGCCAACA |  |
|  | IAPV6249F | GGTGTCGAGGAGGACTTGAC |  |
|  | T7-13LF | ---------------------------------------- | Amplification of full length IAPV genome |
|  | IAPV9613R | TTTTTTTTTTTTTTTTTAAATTTACCTAATTCGAAAATTTTG |  |
|  | qIAPV-F7965 | CCAGCCGTGAAACATGTTCTTACC | real-time RT-PCR primers for quantitation of all IAPV loads |
|  | qIAPV-R8191 | ACATAGTTGCACGCCAATACGAGAAC |  |
| CBPV | CBPV-1LF | TAATACGACTCACTATAGGGTAAACTTTAGGACTAAGATGAATC | Amplification of fragment CBPV RNA1-1 of CBPV RNA1 |
|  | CBPV2490R | ACTACTAGAAACTCGTCGCTTCG |  |
|  | CBPV2471F | AGCGACGAGTTTCTAGTAGTAGTGG | Amplification of fragment CBPV RNA1-2 of CBPV RNA1 |
|  | CBPV-1LR | CTACCTTACCAGTGCCTGACGGACTTATC |  |
|  | CBPV-2SF | TAATACGACTCACTATAGGGTAAACCTTAGGCTTTCATC |  |
|  | CBPV-2LR | AACCATGATAGGGCGTATGACGACTTATCTATC |  |
|  | pACYC1905F | CCCTATAGTGAGTCGTATTAAGTAATACAAGGGGTGTTATGA | Amplification of linearized vector pACYC177 for RNA1 |
|  | CB3655-P1580R | GTCAGGCACTGGTAAGGTAGATACTGGCTTACTATGTTGG |  |
|  | pACYC-1905F | CCCTATAGTGAGTCGTATTAAGTAATACAAGGGGTGTTATGA | Amplification of linearized vector pACYC177 for RNA2 |
|  | CB2286-P1580R | TCATACGCCCTATCATGGTTATACTGGCTTACTATGTTGG |  |
|  | pACYC1567F | GACACCCTCATCAGTGCCAACA |  |
|  | pACYC2178R | GCTTGATGGTCGGAAGAGG |  |
|  | CBPV-1SF | TAATACGACTCACTATAGGGTAAACTTTAGGACTAAGATG | Amplification of CBPV RNA 1 with T7 promoter |
|  | CBPV-1LR | -------------------------------------------------------------------- |  |
|  | CBPV-2SF | TAATACGACTCACTATAGGGTAAACCTTAGGCTTTCATC | Amplification of CBPV RNA 2 with T7 promoter |
|  | CBPV-2LR | AACCATGATAGGGCGTATGACGACTTATCTATC |  |
